# Uncommon cause of fever in a child with steroid-dependent nephrotic syndrome 

**DOI:** 10.5414/CNCS110062

**Published:** 2020-07-07

**Authors:** Sidharth Kumar Sethi, Shyam Bansal, Ronith Chakraborty, Rahul Jain, Nikita Wadhwani, Rupesh Raina

**Affiliations:** 1 Kidney Institute, Medanta, The Medicity Hospital, Gurgaon, Haryana, India, and; 2 Akron General Medical Center, and; 3 Akron Children’s Hospital, Akron, OH, USA

**Keywords:** CMV disease, fever of unknown origin, mixed opportunistic infection, Mycoplasma pneumoniae, steroid-dependent nephrotic syndrome

## Abstract

Background: Children with nephrotic syndrome are vulnerable to developing infections due to a state of relative immunodeficiency, malnourishment, and use of immunosuppression. Case characteristics: We herein report the case of a 3-year-old child with steroid-dependent nephrotic syndrome who presented to us with fever of unknown origin. Observation: The child was found to have an atypical mixed infection with mycoplasma and cytomegalovirus. Outcome: The infection completely resolved with appropriate treatment and lowering of immunosuppression. Message: Persistently febrile pediatric patients, especially in the setting of recent immunosuppression and absence of otherwise-identified infectious pathogens, should be screened for atypical mixed infections.

## Background 

Children with nephrotic syndrome (NS) are vulnerable to developing a myriad of infections due to their state of relative immunodeficiency and malnourishment caused by urinary losses of immunoglobulins and albumin as well as the use of potent immunosuppressants. The incidence of infectious complications in this cohort has been reported to vary between 32 and 38% in different studies [1, 2, 3, 4, 5, 6, 7, 8]. The spectrum is versatile but frequently includes spontaneous bacterial peritonitis, septicemia, urinary tract and skin infections, upper and lower respiratory tract infections, meningitis, and lastly, viral infections (herpes simplex virus, varicella-zoster virus, measles) [6, 7]. Apart from increasing the morbidity and mortality, infections also induce relapses of NS and steroid resistance [8]. The incidence and frequency of serious infections has been shown to be higher in the setting of steroid-dependent NS, frequently-relapsing and steroid-resistant cases of nephrotic syndrome [8, 9]. We herein present a 3-year-old steroid-dependent child with NS with an uncommon mixed infection following a prolonged immunosuppressive treatment regimen. 

## Case report 

A 3-year-old girl presented at our clinic with daily remittent fever of 2 weeks’ duration that was unresponsive to antipyretics. She was experiencing high-grade fever, fluctuating between 38 and 40 °C and occurring up to 3 times a day. Her past medical history was significant for NS diagnosed 1 year prior. She was started on a standard immunosuppressive regimen consisting of prednisolone (9 mg on alternate days). She could not be weaned off steroids despite repeated trials and required a high-dose regimen to stay in remission. In view of her high-threshold steroid dependence, the parents were counselled regarding the use of oral cyclophosphamide or mycophenolate mofetil (MMF) therapy. With parental consent, she was started on MMF (1,000 mg/m^2^/day). The patient was subsequently lost to follow-up. At the time of presentation, the child was in remission with no proteinuria and with normal serum albumin levels. 

Four months into treatment for NS, she developed remittent bouts of high fever for 2 weeks, after which she returned at our hospital seeking medical care. After reviewing her medical records, it was determined that the patient was on both MMF (1,000 mg/m^2^/day) and prednisolone (9 mg on alternate days). She had been noncompliant with the prescribed corticosteroid taper. Her local clinician did not taper the initial prescription of 9 mg alternate-day steroid (her weight being 20 kg). On admission, she was noted to have hepatosplenomegaly and leucocytosis (Table 1). Empiric treatment was commenced with intravenous ceftriaxone and stress doses of prednisolone. At presentation, two sets of blood and urine cultures were sent for analysis, and they reported no growth. 

**Figure 1.**
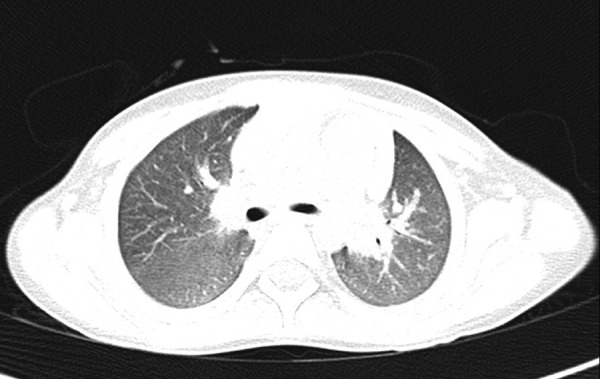
Computed tomography scan of chest and abdomen show hepatosplenomegaly and diffuse ground glass haze in both lung fields.

Serologic testing for dengue, malaria, typhoid, and rickettsia were non-reactive. Her serum ANA (anti-nuclear antibodies) and complement protein (C3, C4) levels were normal. The child had a normal nutrition state, with both height and weight percentiles being at 50^th^ percentile for age and sex as per Centers for disease control and prevention (CDC) standards. A transthoracic echocardiogram failed to demonstrate any vegetation suggestive of infective endocarditis, and the chest X-ray was normal. Ultrasound of the abdomen showed no abnormality apart from hepatosplenomegaly. A pediatric hematology review was obtained, and a bone marrow aspiration study was undertaken, which showed cellular, reactive bone marrow with normal hematopoietic cells, and no evidence of leukemia or hemophagocytosis. Computed tomography scan of chest and abdomen showed hepatosplenomegaly and diffuse ground glass haze in both lung fields (Figure 1). 

On further investigation, she was found to have raised IgG and IgM antibody titers (17.4 AU/mL and 27 AU/mL, respectively: normal value < 10 AU/mL) to *Mycoplasma pneumoniae*. A repeat sample during the convalescent period showed a 4-fold rise in IgG titer against *Mycoplasma pneumonia*, thus confirming the infection. Subsequent addition of azithromycin was followed by a remarkable improvement in her clinical condition. A quantitative viral PCR assay for Ebstein-Barr virus (EBV)DNA was negative. PCR testing for cytomegalovirus (CMV)****DNA revealed 56,340 copies/mL, and her MMF was stopped. Within a week of instituting intravenous ganciclovir therapy, the viral load declined to 180 copies/mL. A repeat PCR assay 1 week later was negative for CMV DNA. She was finally switched to oral valganciclovir and was completely asymptomatic at the time of discharge with undetectable viremia, normal leucocyte count, and absence of organomegaly. Currently, the child has been in remission for the last 6 months, without immunosuppressive therapy. 

## Discussion 

Opportunistic infections occur in the setting of intensified immunosuppression involving high-dose steroids, antimetabolites, and biologics. MMF is a new immunosuppressant recently being used as a steroid-sparing agent in pediatric steroid-sensitive NS. It has now been used in multiple retrospective studies and a recent randomized controlled trial [10, 11, 12]. Fever is a common manifestation of CMV disease, but an infrequent cause of fever of unknown origin (FUO) [13]. The incidence of CMV antigenemia increases manifold with the use of combination immunosuppression. Apart from FUO, it can also present with a mononucleosis-like picture, but more serious complications including CMV colitis or pneumonitis may also result in immunocompromised patients (especially oncology and post-transplant patients) [1, 2]. 

CMV should be suspected in FUO patients in the presence of otherwise unexplained leukopenia, relative lymphopenia, thrombocytopenia, atypical lymphocytosis, or transaminitis. Prompt CMV testing using viral load assay should be sought in such cases. The presence of typical cytopathic changes associated with CMV in tissue specimens or a very high serum IgM titer (in the absence of rheumatoid factor) can also suffice to establish a diagnosis [14]. 

*Mycoplasma pneumoniae* has been sparsely cited as a cause of FUO [15, 16, 17]. The clinical course of mycoplasma infection in immunosuppressed patients may follow a variable pattern, and a prolonged illness may result. Immunocompromised patients are more likely to present with pneumonia as compared with upper respiratory tract infection or bronchiolitis, which are commonly encountered among immunocompetent patients. The clinical manifestations, however, appear to be similar, and these include fever, malaise, and cough with no or mild sputum production [18, 19, 20, 21, 22]. It has been shown to cause varied extra-pulmonary involvement in the form of septic arthritis, cerebellar ataxia, Guillain-Barre syndrome, transverse myelitis, and aseptic meningitis [23, 24, 25]. 

*Mycobacterium tuberculosis*, a well-known cause of FUO in both normal and immunocompromised patients, should also be considered early in the course of disease in patients coming from areas with high endemicity. Fungal opportunistic infections, yet another cause of morbidity in immunocompromised population, are not a frequent cause of FUO, with the exception of histoplasma, which has been reported to result in prolonged febrile illness with subacute pulmonary symptoms in several studies. Common non-infectious causes like malignancies, adrenal insufficiency, vasculitides, and drug fever can also lead to FUO and should be considered during the work-up [26, 27, 28]. 

## Conclusion 

We suggest that persistently febrile pediatric patients, especially in the setting of recent immunosuppression and absence of otherwise-identified infectious pathogens, should be screened for atypical mixed infections like CMV and mycoplasma. 

## Funding 

There is no funding to report. 

## Conflict of interest 

There is no conflict of interest. 

**Table 1.** Lab investigations.

Hemoglobin (g/dL)	13.5 (12 – 15)
TLC (×10^9^/mL)	14500 (4 – 11)
Platelets (×10^9^/mL)	456 (150 – 400)
Creatinine (mg/dL)	0.4 (0.6 – 1.2)
BUN (mg/dL)	16 (7 – 20)
ALT (U/L)	22 (8 – 20)
AST (U/L)	20 (8 – 20)
ALP (U/L)	154 (30 – 100)
C-reactive protein (mg/L)	5.7 (0 – 10)
Quantitative DNA PCR	
EBV	Negative
CMV	56,340 copies/mL
Anti-nuclear antibodies	Non-reactive
Anti-mycoplasma antibodies (AU/mL)	
IgG	17.4 (< 10)
IgM	27 (< 10)
Blood and urine cultures	Sterile
Chest X-ray	Normal
Ultrasound abdomen	Hepatosplenomegaly

ALP = alkaline phosphatase; ALT = alanine aminotransferase; AST = aspartate aminotransferase; BUN = blood urea nitrogen; CMV = cytomegalovirus; EBV = epstein-barr virus; TLC = total leukocyte count.
